# Improved Speech Hearing in Noise with Invasive Electrical Brain Stimulation

**DOI:** 10.1523/JNEUROSCI.1468-21.2022

**Published:** 2022-04-27

**Authors:** Prachi Patel, Bahar Khalijhinejad, Jose L. Herrero, Stephan Bickel, Ashesh D. Mehta, Nima Mesgarani

**Affiliations:** ^1^Mortimer B. Zuckerman Mind Brain Behavior Institute, Columbia University, New York, New York 10027; ^2^Department of Electrical Engineering, Columbia University, New York, New York 10027; ^3^Hofstra Northwell School of Medicine, New York, New York 11549; ^4^Feinstein Institute for Medical Research, New York, New York 11030

**Keywords:** auditory cortex, human electrophysiology, speech preception

## Abstract

Speech perception in noise is a challenging everyday task with which many listeners have difficulty. Here, we report a case in which electrical brain stimulation of implanted intracranial electrodes in the left planum temporale (PT) of a neurosurgical patient significantly and reliably improved subjective quality (up to 50%) and objective intelligibility (up to 97%) of speech in noise perception. Stimulation resulted in a selective enhancement of speech sounds compared with the background noises. The receptive fields of the PT sites whose stimulation improved speech perception were tuned to spectrally broad and rapidly changing sounds. Corticocortical evoked potential analysis revealed that the PT sites were located between the sites in Heschl's gyrus and the superior temporal gyrus. Moreover, the discriminability of speech from nonspeech sounds increased in population neural responses from Heschl's gyrus to the PT to the superior temporal gyrus sites. These findings causally implicate the PT in background noise suppression and may point to a novel potential neuroprosthetic solution to assist in the challenging task of speech perception in noise.

**SIGNIFICANCE STATEMENT** Speech perception in noise remains a challenging task for many individuals. Here, we present a case in which the electrical brain stimulation of intracranially implanted electrodes in the planum temporale of a neurosurgical patient significantly improved both the subjective quality (up to 50%) and objective intelligibility (up to 97%) of speech perception in noise. Stimulation resulted in a selective enhancement of speech sounds compared with the background noises. Our local and network-level functional analyses placed the planum temporale sites in between the sites in the primary auditory areas in Heschl's gyrus and nonprimary auditory areas in the superior temporal gyrus. These findings causally implicate planum temporale in acoustic scene analysis and suggest potential neuroprosthetic applications to assist hearing in noise.

## Introduction

Speech communication often takes place in the presence of competing sound sources, yet we are able to carry on conversations effortlessly even when speech signals are mixed with considerable noise. The neural processes that enable the segregation and enhancement of the acoustic features of speech relative to background noise remain largely unknown (Bregman, 1994; Assmann and Summerfield, 2004). It has been shown that the invariance of speech emerges gradually as the neural responses to sound ascend in the auditory pathway from the cochlea (Wang and Shamma, 1994) to the thalamus (Miller et al., 2001) and primary and nonprimary cortical areas (Kumar et al., 2007; Hong et al., 2008; Rabinowitz et al., 2013; Mesgarani et al., 2014). Studies of speech-in-noise perception in humans have found an increasingly invariant cortical representation of speech in the nonprimary auditory cortex that is unaffected by noise (Kell and McDermott, 2019). This invariant representation is constructed by the selective suppression of the encoded noise features in neural responses that occur across multiple anatomic areas, including Heschl's gyrus (HG), the planum temporale (PT), and the superior temporal gyrus (STG) (Khalighinejad et al., 2019). The causal role of each of these regions in removing noise from speech is not yet fully understood; and in particular, the role of the PT in acoustic scene analysis remains debated (Hickok and Saberi, 2012).

Anatomical studies of the PT have shown that it is an intermediary area between HG and the STG (Griffiths and Warren, 2002; Morosan et al., 2005; Hickok and Saberi, 2012), placing its function between those of nonspecific responses to speech in HG and speech-specific responses in the lateral STG (Humphries et al., 2014). These findings are consistent with the functional studies that have speculated on the role of the PT in the separation and identification of sound sources from background sounds (Griffiths and Warren, 2004; Isenberg et al., 2012). Indeed, an increased cortical surface area in the PT has been shown to correlate with the accuracy of acoustically distorted speech sound categorization (Elmer et al., 2013), confirming the role of the PT as a primary processor of rapidly changing acoustic cues in speech and nonspeech signals (Meyer et al., 2012).

Beyond these correlative studies that suggest a role for the PT in processing speech in noise, the causal role of the PT in speech perception in noise has not yet been studied. This absence of progress is because of the lack of methods that can manipulate neural responses with a high degree of spatiotemporal specificity in awake behaving humans as they engage in realistic speech-in-noise perception tasks. One such method that is often used for clinical mapping is electrical brain stimulation (EBS). During EBS, an electrical charge is delivered to a focal brain area to disrupt its function (Parvizi and Kastner, 2018). EBS in humans is unique because humans can articulate their perceived experiences during stimulation, an advantage that is absent in animal models. In the auditory domain, previous studies have reported that EBS of the STG causes auditory hallucinations (e.g., hearing a buzzing sound), illusions, and errors (e.g., hearing a distorted version of a sound) (Selimbeyoglu and Parvizi, 2010; Leonard et al., 2019), suppression of tinnitus (Fenoy et al., 2006), transient hearing loss (Sinha et al., 2005), and have implicated the posterior STG in basic auditory functions, such as syllable discrimination (Boatman et al., 1995; Miglioretti and Boatman, 2003). On the other hand, EBS of the medial HG can induce the perception of distinct tones (Donovan et al., 2015). The effect of EBS in PT remains less clear because of the limited intracranial studies with depth coverage to target this region. Notably, none of the previous EBS studies in the human auditory cortex has demonstrated a facilitative role for EBS in speech perception, a task that remains challenging, particularly in adverse and noisy acoustic conditions.

In this study, we used a unique instance in which an epilepsy patient was implanted with both grid and depth electrodes (intracranial EEG [iEEG]) covering multiple auditory areas, including HG, the PT, and the STG. We stimulated and recorded the responses of HG, the PT, and the STG during a speech-in-noise perception task. Stimulation of the PT sites resulted in significant improvement in both the perceived quality and intelligibility of speech. Furthermore, we showed distinct local- and network-level properties for these neural sites using spectrotemporal receptive field analysis, corticocortical evoked potentials (CCEPs), and speech-nonspeech discriminability. This study suggests that the PT has a causal role in speech-in-noise perception and suggests potential neuroprosthetic solutions to enhance speech-in-noise perception, which currently remain a major challenge for individuals with hearing loss.

## Materials and Methods

### Experimental design and statistical analysis

#### Participants and data collection

A 27-year-old female adult with pharmacoresistant focal epilepsy was the subject of this study. The subject had self-reported normal hearing and showed no difficulty in speech communication during the neuropsychological language tests done before implant. The subject underwent chronic iEEG monitoring at North Shore University Hospital to identify epileptogenic focus in the brain for later removal. She was implanted with both surface grid & strip as well as depth electrodes (PMT). Electrodes showing any sign of abnormal epileptiform discharges, as identified in the epileptologists' clinical reports, were excluded from the analysis. All included iEEG time series were manually inspected for signal quality and were free from interictal spikes. All research protocols were approved and monitored by the institutional review board at the Feinstein Institute for Medical Research, and informed written consent to participate in research studies was obtained from the subject before the implantation of electrodes.

#### Stimulus

The subject participated in four experiments: EBS, listening to continuous speech, listening to speech versus nonspeech sounds, and CCEPs. The details of each experiment are described below.

### Experiment 1: EBS during speech-in-noise

Bipolar electrical stimulation was delivered to neighboring contacts along the shaft of a stereotactic high-density depth electrode (16 contacts, 2.2 mm intercontact distance, 1.3 mm contact size) using 200 µs squared-wave biphasic pulses at 50 Hz for 4 s (S12 stimulator, Grass Technologies). Two intensities (1 and 3 mA) were tested. The patient was asked to keep her eyes open during the procedure. We used a standard speech-in-noise intelligibility test (BKB-SIN) (Wilson et al., 2007) to measure the intelligibility of speech during stimulation of electrodes. The speech-in-noise sentences were sampled at 20,000 Hz.

**Figure 1.**
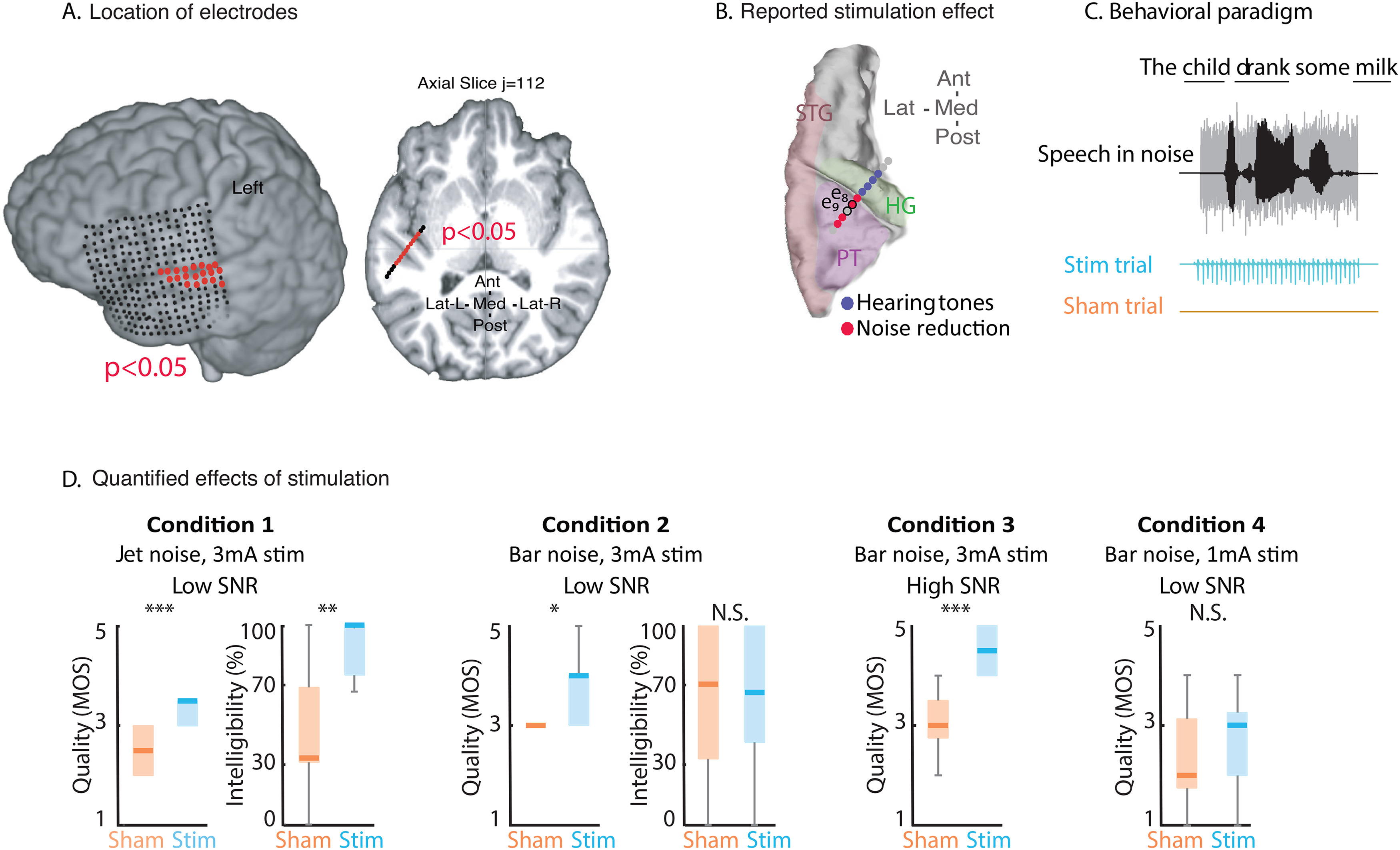
Perceptual reduction in background noise by EBS. ***A***, The anatomic location of the depth and grid electrodes. ***B***, Behavioral report of the subject when bipolar, 50 Hz electrical stimulation was delivered to electrode pairs. The subject reported hearing tones when the blue electrodes (located in HG) were stimulated and reported background noise reduction when the red electrodes (located in the PT) were stimulated. ***C***, Experimental design used to measure the subject's perceptions of the quality and intelligibility of speech in background noise during EBS. The subject heard sentences from the BKB-SIN speech intelligibility corpus in background noise, played either in sync with an electrical stimulation pulse train (stim trial, green) or with no electrical stimulation (sham trial, gray). An example sentence and the keywords used to calculate the intelligibility score are underlined. ***D***, Perceived quality of speech (mean opinion score) in sham and stim trials under four conditions: (1) jet noise with a low SNR and 3 mA stimulation current; (2) bar noise with a low SNR and 3 mA stimulation current; (3) bar noise with a high SNR and 3 mA stimulation current; and (4) bar noise with a low SNR and 1 mA stimulation current. Middle line indicates median. Box represents quartiles. Whiskers represent maximum and minimum.

Although every contact was stimulated at least once, the objective and subjective measurements of the sham versus stimulation trials were calculated based on 60 stimulation trials of one site along electrode shaft in the left PT (see Fig. 1*B*, electrode contact 8-9) because they had the highest noise reduction effect based on subject self-report. Randomized sham stimulations (asking the patient to report the percepts without introducing any current into the electrodes) were performed for the quantification of intelligibility and quality improvement. During the sham trials, we specifically tested the placebo effect by counting “1, 2, 3” before clicking the stimulator button, similar to how the stim trials were performed, but the electrical current was set at 0 mA. EEG and clinical signs in the patient ruled out the involvement of HG, the PT, and the STG in the patients' seizures, and the patient was instructed to report whether the electrical stimulation caused her typical seizure auras. The patient did not report experiencing seizure auras during the experiment.

### Experiment 2: listening to continuous speech stories

The subject listened to stories recorded by four voice actors (two males and two females) with a duration of 20 min and a sampling rate of 11,025 Hz. The sentences were on an average 5.2-s-long with intersentence interval of 0.5 s. The stimuli were presented using a single Bose SoundLink Mini 2 speaker situated directly in front of the subject. The stimuli and the neural responses to these stimuli were used to calculate the spectrotemporal receptive fields of each neural site.

### Experiment 3: listening to speech versus nonspeech sounds

The subject listened to 20 min of 69 commonly heard natural sounds. Among these 69 sounds were 16 samples of English and foreign speech and 53 nonspeech sounds from 14 categories (coughing, crying, screaming, music [jazz, pop, classical], animal vocalizations, laughing, syllables, sneezing, breathing, singing, shooting, tones, drumming, and subway noise) (Khalighinejad et al., 2021). The sounds were on an average 12.5-s-long with intersentence interval of 0.5 s. To find the neural representation of each sound, we averaged the high γ (70-150 Hz) activity in response to the sound over time. Data from this experiment were used for the latency analysis (see Fig. 5) and for the speech/nonspeech analysis (see Fig. 6). The latency was measured as the excitatory peak of spectrotemporal receptive field (STRF) as the center of gravity along the time dimension. The STRF for each electrode was obtained using all the sounds (speech and nonspeech).

### Experiment 4: CCEPs

CCEP mapping was performed with bipolar stimulation of each pair of adjacent electrodes with single pulses of an electrical current (10 mA, biphasic, 100 µs/phase, 30 trials per electrode pair) using a Grass S12 cortical stimulator. The interstimulation interval was 1.5 s (±0.5 s jitter). The current magnitude of 10 mA was chosen for the grid/strip electrodes and 4 mA for depth electrodes. These values were chosen as they were the maximum current that did not induce epileptiform discharges in areas outside of the seizure onset zone. The patient was awake and at rest at the time of CCEP recording.

#### Preprocessing neural data

iEEG signals were acquired continuously at 3 kHz per channel (16-bit precision, range ± 8 mV, DC) with a data acquisition module (Tucker-Davis Technologies). The skull electrodes were used as references, as dictated by the recording quality at the bedside after online visualization of the spectrogram of the signal. Speech signals were recorded simultaneously with the iEEG for subsequent offline analysis. All further processing steps were performed offline. The iEEG data were resampled to 512 Hz. A first-order Butterworth high-pass filter with a cutoff frequency of 1 Hz was used to remove DC drift. Line noise at 60 Hz and its harmonics (up to 240 Hz) were removed using second-order IIR notch filters with a bandwidth of 1 Hz. The envelope of high γ activity, which correlates with neural firing in the proximity of the electrodes (Ray and Maunsell, 2011; Buzsáki et al., 2012), was used as a measure of the neural response. To obtain the envelope of this broadband signal, we first filtered the data into eight frequency bands between 70 and 150 Hz (Edwards et al., 2009). Then, the envelope of each band was obtained by taking the absolute value of the Hilbert transform. We took the average of all eight frequency bands as the final envelope. The data were resampled to 100 Hz for further analysis. Neural sites that were responsive to sound were determined by a *t* test between responses to silence and all sounds from Experiment 3 (*t* test, FDR-corrected, *q* < 0.01) (Benjamini and Yekutieli, 2001).

Video-iEEG monitoring demonstrated seizures with an onset at the medial and inferior temporal lobe. Frequent interictal spike and wave discharges were also observed in the posterior subtemporal regions. None of the electrodes in HG, the PT, or the STG were part of the irritative or seizure onset zones.

#### Behavior quantification

To evaluate the quality of speech sentences, we asked the subject to repeat the sentence that was played and rate its quality using the Mean Opinion Scale (MOS) scale (Salza et al., 1996). An MOS rating of between 1 and 5 (1= bad; 2 = poor; 3 = fair; 4 = good; 5 = excellent) was given. Intelligibility was measured as the percentage of key words identified in presented sentences using the BKB-SIN test (Wilson et al., 2007).

To evaluate the perceived tone frequency when HG electrodes were stimulated, the subject was given a knob that varied the tone frequencies being played. The subject was asked to move the knob until the presented pitch matched the tone frequency she perceived when the HG electrode was stimulated.

#### STRFs

STRFs were computed by a normalized reverse correlation algorithm (Theunissen et al., 2001) using STRFLab (Theunissen et al., 2001). We first converted the sound waveform into a time-frequency representation using a cochlear frequency analysis model consisting of a bank of 128 asymmetric constant-Q filters spaced equally along a logarithmic axis (Chi et al., 2005). The MATLAB code used to calculate the auditory spectrogram is available at http://nsl.isr.umd.edu/downloads.html. The output of the filter bank was then resampled to 16 frequency bands to prevent parameter overfitting. The amplitude of the high γ band was used as the measure of neural activity. Regularization and sparseness parameters were found via cross-validation (David et al., 2007). The best frequency and response latency parameters were estimated by finding the center of the excitatory region of the STRF along the frequency and time dimensions.

#### Multidimensional scaling (MDS) analysis

To calculate the MDS diagram of speech and nonspeech sounds, we first found the average of the high γ activity across the duration of the sounds in each category. In determining these averages, we eliminated the small segments of silences during the trial by averaging only the samples with above-threshold spectrogram energy. Next, we calculated the Euclidean distance between the average response to different sounds to find a dissimilarity matrix. To visualize this dissimilarity matrix, we used a two-dimensional MDS algorithm based on Kruskal's normalized criterion to minimize stress for the two MDS dimensions (Cox and Cox, 2008).

#### Analysis of CCEPs

CCEP mapping was performed with bipolar stimulation of each pair of adjacent electrodes with single pulses of electrical current (10 mA, biphasic, 100 µs/phase, 30 trials per electrode pair) using a Grass S12 cortical stimulator (Grass Technologies). Electrical stimulation artifact was found in first 20 ms of the data. This artifact was removed from each CCEP before analysis. Recorded neural data were sampled at 3000 Hz and bandpass filtered at 0.1-1 kHz. Acquired data were notch filtered at 60 Hz. The interstimulation interval was 1.5 s (±0.5 s). Differences in the interstimulation interval had no effect on evoked potentials. A current magnitude of 10 mA was chosen for grid/strip electrodes and 4 mA for depth electrodes. CCEPs in the human cortex generally consist of an early sharp response (10-50 ms poststimulation) and a later slow wave (50-250 ms). However, it has been shown that polarity can be positive in some cases, and there is a similar correlation between CCEPs and resting when the N1 or P1 response is used (Keller et al., 2011). Therefore, to compare the connectivity among regions, we used the absolute value of the response during the duration of 20-30 ms, which corresponds to the N1 component (Keller et al., 2014a).

#### Graph analysis of CCEPs

For each stimulation and response site, mean evoked potentials (derived from 20 repetitions) were normalized relative to the mean and SD of the prestimulus baseline (−500 to −5 ms). To assess which electrodes are close to which other electrodes, an adjacency matrix was formed, with rows indicating stimulated sites and columns indicating CCEP recording sites. The maximum absolute value of the N1 component of the normalized CCEP between two sites was used to fill in the values of the adjacency matrix. A *Z* score of 12 was determined as a cutoff to convert the adjacency matrix to a sparse matrix of 0 s and 1 s that indicated whether the corresponding row electrode was connected to the corresponding column electrode. We did not find any difference in the results when a different threshold within the range of 5-15 was used. To evaluate electrode connectivity, specifically the directions of connections and relative node connectivity, digraphs were made from this adjacency matrix using the MATLAB function *digraph*. The electrode locations on the graph were obtained by using the MATLAB function *layout* with the parameter *force*, which estimates node coordinates based on the force-directed structure of the graph (Fruchterman and Reingold, 1991). This relative location of nodes was determined by the force-directed layout using node connectivity in the adjacency matrix, such that nodes that are not connected are placed farther apart in the plot and those that are connected are placed close together. Finally, the flow of information was assessed by measuring the shortest path in the digraphs using the function *shortestpath* with Dijkstra's algorithm.

## Results

### Direct electrical stimulation of auditory areas elicited hallucinations of tones and noise reduction effects

A 27-year-old patient with refractory epilepsy was implanted with intracranial depth and grid electrodes to localize the source of seizure activity. The positions of intracranial electrodes covering the perisylvian regions are shown on the subject's neuroanatomical space with the speech responsive sites (see Materials and Methods) in red (Fig. 1*A*). In Figure 1*B*, the anodes of the eight bipolar contacts that generated an auditory effect with bipolar electrical stimulation are shown. Stimulation of the four electrodes in the left HG induced an auditory hallucination effect, and the patient matched the induced frequencies to 230, 230, 250, and 250 Hz. Stimulation of the three electrodes in the left PT and one electrode on the boundary of the left PT and HG generated a noise-suppression effect, meaning that the patient reported an increase in the volume and clarity of speech compared with background noise. In the following section, we describe the behavioral task and subjective and objective measures we used to systematically evaluate the improved quality and intelligibility of speech in noise.

### Measurement of improved intelligibility and quality of perceived speech after EBS of the PT electrode

We systematically tested the behavioral effect observed during clinical stimulation while the subject listened to speech in noise sentences (see Materials and Methods). Half of the trials were chosen randomly for simulation (stim trials), and the other half were performed with no stimulation (sham trials) (Fig. 1*C*). The patient was asked to repeat the sentence and rate its quality using the MOS scale from 1 (poor) to 5 (excellent) (Salza et al., 1996) (see Materials and Methods). The reported MOS scale was used as the measure of perceived quality of speech, and the percentage of correctly repeated key words was used as the measure of perceived intelligibility of speech (Wilson et al., 2007).

We tested four different conditions (Fig. 1*D*). In Condition 1, we used jet background noise; in Conditions 2, 3, and 4, we used bar background noise. In Condition 4, we reduced the stimulation current from 3 to 1 mA (Fig. 1*D*). In Condition 1, where the background noise was jet noise with a signal-to-noise ratio (SNR) of −2.27 dB, the patient rated the speech quality significantly higher in the stim trials than in the sham trials (MOS = 3.2 vs 2.4, *p* < 10^–3^, *N* = 24, Wilcoxon rank-sum test). In addition, speech intelligibility was significantly higher in the stimulated jet noise condition than in the sham trials (91% correct vs 46% correct, *p* < 0.005, *N* = 24, Wilcoxon rank-sum test).

In Condition 2, where the background noise was bar noise with an SNR of −2.9 dB, the patient rated the speech quality significantly higher in the stim trials than in the sham trials (3.7 vs 3.1, *p* = 0.02, *N* = 36, Wilcoxon rank-sum test). However, we did not find a significant difference between the intelligibility of speech in the stim trials compared with the sham trials (67% correct vs 65% correct, *p* = 0.9 [not significant], *N* = 36, Wilcoxon rank-sum test).

In Condition 3, we tested the effect of stimulation in speech in noise with a high SNR, where the background noise was bar noise with an SNR of 3 dB. All of the sentences in this high-SNR condition were intelligible with or without stimulation. However, the perceived quality of the speech was significantly higher in the stim trials than in the sham trials (4.5 vs 3, *p* < 10^–3^, *N* = 41, Wilcoxon rank-sum test).

In Condition 4, as a control condition, we reduced the stimulation current to 1 from 3 mA (with bar background noise and an SNR of −4 dB). We observed that stimulation at a lower current (1 mA) did not result in improvement in the perceived quality of speech, although the patient could detect that a lower stimulation current was being used (*p* = 0.57, Wilcoxon rank-sum test).

### Description of the patient's experience during stimulation

Subjective reports of the effects of electrical stimulation are shown in Table 1 (see also Movie 1). Stimulation of electrode contacts 8-9 in the PT caused the patient to experience a complex auditory effect: she reported improved quality and intelligibility of speech in background noise. In her own words, “The voices get a lot more clear; I still hear the noise, but the voice gets a lot more clear”; “It is always the speech that goes stronger; it's never the background noise”; “If two people talk at the same time, they both increase; it only happens for the background noise”; and “[It's] as if you take an equalizer for music and change the mode of how you want to hear.” In Table 1, the patient's description of 1 versus 3 mA stimulation is also included; she could feel the stimulation in both cases, but only the 3 mA stimulation caused the noise reduction effect.

**Table 1.** The full subjective report of the stimulation experiment

Patient describing the denoising effect of stimulation:	
I heard the noise when it started, and as soon as you did whatever you did, all I heard was his voice.	
The voices get a lot clearer. I still hear the noise, but the voice gets a lot clearer, as if someone is saying it in my ear. I still hear the background, but the voice is louder, more prominent.	
It is always the speech that gets stronger; it's never the background noise. The person's voice becomes more prominent over the background noise, as if someone gives that person a microphone. Kind of like they tell everyone to hush, to turn their background noise down and let this person talk. When you are both talking, I hear you both just as clear; it is just the noise. If two people talk at the same time, they both increase; it's just the background noise.	
The easiest way for me to explain it is when you take an equalizer for music and you change the different modes of how you want to hear the music; that's how.	
Patient describing the time-varying nature of the denoising effect:	
I heard “snow falls” clearly, and there were two more words, but I didn't hear the two words. I heard “snow falls” at approximately 5 (excellent), and then after that, I didn't hear the two other words. I just feel that it all blended in as the background noise came back.	
I heard [the first word] and I felt that something happened, but the quality started to fade. It was first at 4 (good), and then I heard up to [five], and then I didn't hear anything afterward.	
It is like I hear the voice clearly, and then everything else just comes back along with it, background noise and voices.	
As soon as he started talking, I heard those first two words clearly with no background noise, (without) anything, and then after the word “old,” everything just came back all at once.	
It was a 4 (good) up to the word “mailman dropped,” and then I couldn't understand anything after that. It was 2 (poor) after that because all the background noises came back.	
Half of it (the sentence) was clear; the first half (of the sentence) was clear.	
Patient describing the stimulation effect:	
I can feel kind of like popping in my ear — not a very strong popping, but I can feel change. Like I can feel that something got a bit clearer.	
1 mA vs 3 mA	A lot less strong. Things still got clearer, but not as strong.
	Not feeling it as strong as I heard it before. Still feel it, but less.	

Movie 1. The patient describing her subjective experience during the stimulation and sham trials. 10.1523/JNEUROSCI.1468-21.2022.video.1

### PT sites have distinct spectrotemporal tuning properties

**Figure 2.**
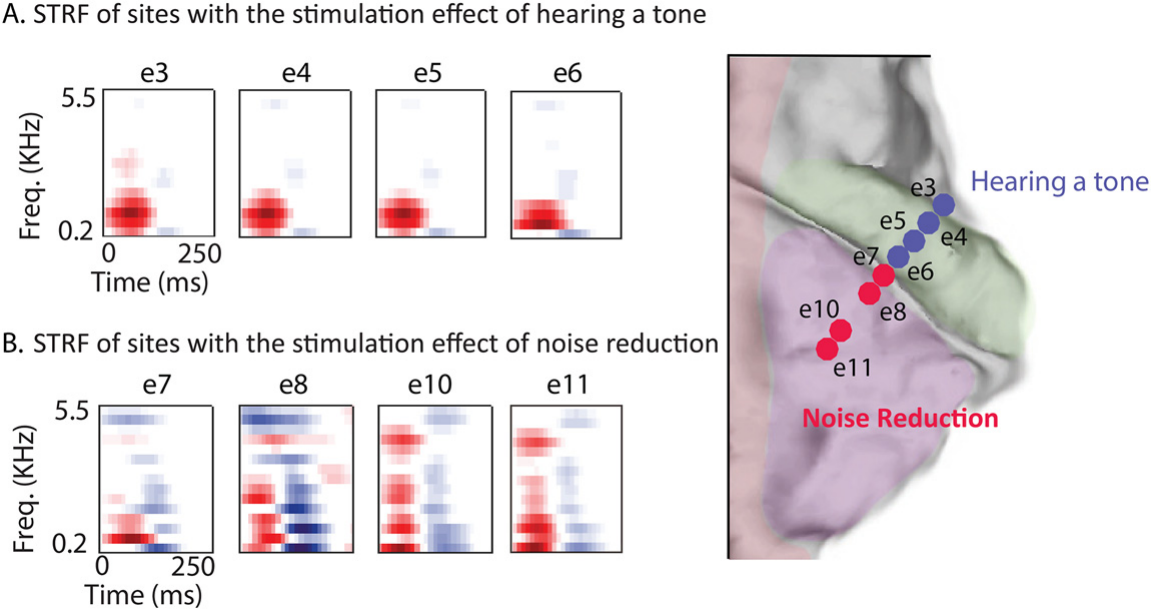
STRFs of neural sites in HG and the PT. ***A***, STRFs of neural sites in HG that generated a perceived tone. ***B***, STRFs of neural sites in the PT that caused a noise reduction effect.

To examine the functional properties of the cortical sites and calculate their temporal and spectral tuning, we calculated the STRF of each electrode. The subject listened to 30 min of clean speech stories without the application of any electrical stimulation. The envelope of the high γ frequency band (70-150 Hz), which correlates with neural firing in the proximity of the electrode (Ray and Maunsell, 2011; Buzsáki et al., 2012), was extracted as the neural response measure of the recorded signals (see Materials and Methods). We found that the electrodes in HG were narrowly tuned to a specific frequency range (Fig. 2*A*); on the other hand, the electrodes in the PT were responsive to temporal changes across a broad range of frequencies, as shown in Figure 2*B*.

It is worth mentioning that we accurately measured the frequency of the hallucinated tone in HG by asking the subject to manually adjust the frequency of a tone generator to the frequency of the induced tone. We did not find any relationship between the best frequency of the contacts in HG and the frequency that was induced through stimulation. Specifically, the subject heard 230, 230, 250, and 250 Hz as a result of stimulation of four contacts in HG with optimal frequencies of 715, 676, 684, and 963 Hz (see Materials and Methods).

### Functional connectivity of HG and the STG through the PT

Stimulation of a neural site affects not only the site where the current is directed but also the network of regions that are connected to it. To examine the spatial extent of the stimulation and to test the differences in connectivity of the cortical sites located in HG and the STG versus the PT and the STG, we used the CCEP method. CCEP mapping applies single pulses of a current to measure electrophysiological responses with accurate localization and evaluate interareal connectivity (Keller et al., 2014a,b, 2018).

Most CCEPs consist of an early (10-30 ms) negative surface deflection labeled N1 and a later (80-250 ms) slow wave labeled N2. Although studies have shown a possibility of shorter N1 component at 1.7 and 2.3 ms (Brugge et al., 2003), the N1 of CCEPs from our study align with the 10-30 ms shown by Keller et al. (2014a). The N1 and N2 components of CCEPs exist across spatially diverse recording sites after stimulation. Therefore, the stimulation-evoked response provides a measure of directional connectivity that is calculated directly from the cortical areas with high spatial resolution. It has been shown that the number of white matter tracts measured with DTI is positively correlated with the strength of the N1 component of CCEPs (Keller et al., 2014a,b, 2018).

**Figure 3.**
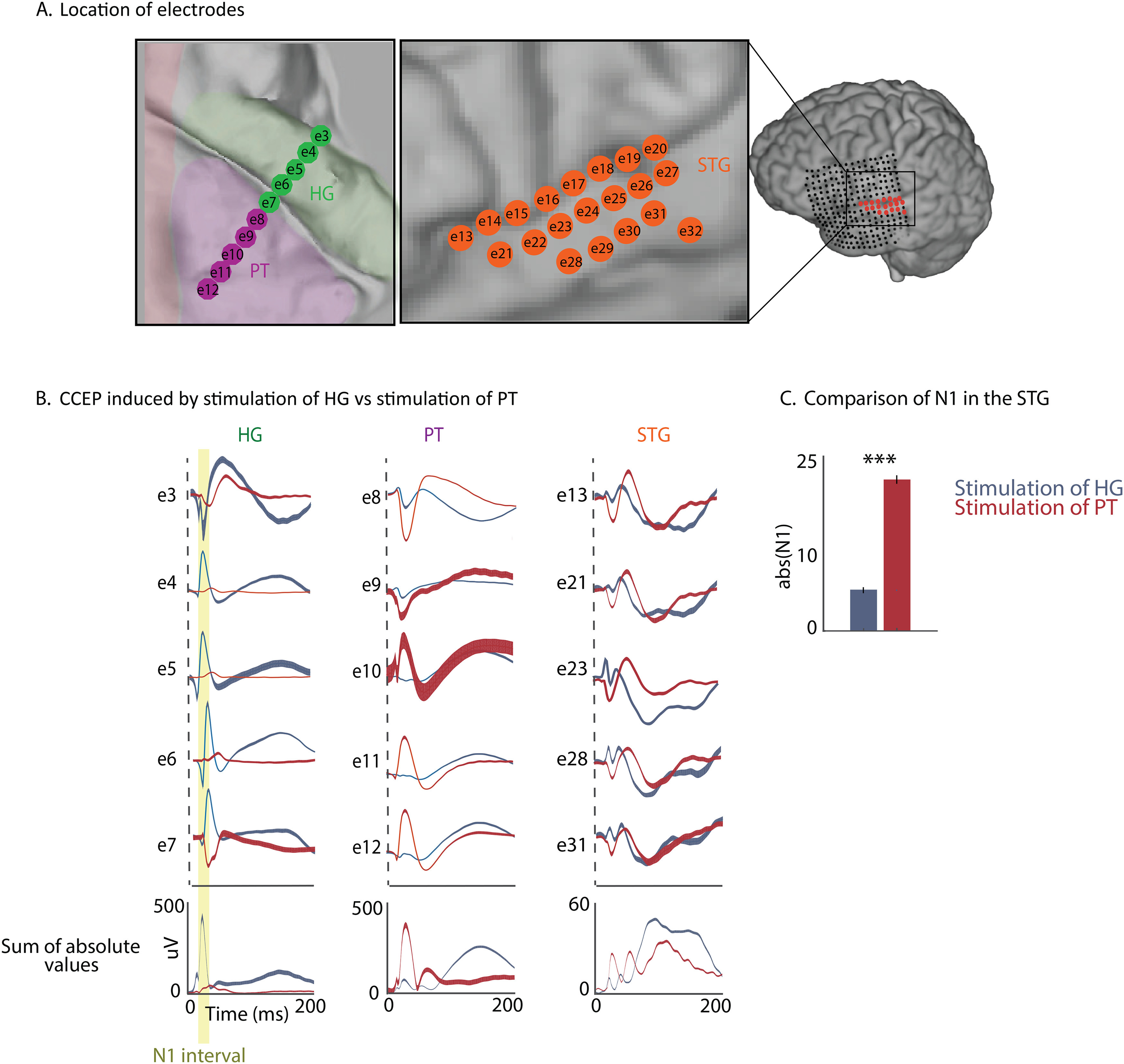
CCEP activity in the STG through stimulation in PT versus HG. ***A***, Location of electrodes in the HG, the PT, and the STG. ***B***, Evoked responses in representative sites in the HG, in the PT, and in the STG using corticocortical stimulation of the HG versus the PT. Bottom row represents the average of the absolute value of the evoked responses for all neural sites in three areas (HG, PT, and STG). ***C***, Comparison of the absolute value of amplitude of N1 recorded in the STG and generated by stimulation of the PT versus HG.

We divided the speech-responsive sites in this subject into three groups based on their anatomic locations: the HG, the PT, and the STG. We tested the connectivity among the sites in HG, the PT, and the STG (Fig. 3*A*). To test whether connectivity between the PT and the STG differs from connectivity between HG and the STG, we examined the N1 component of the evoked responses in the STG induced by stimulation of the PT versus stimulation of HG sites. We observed that the stimulation of electrodes in the PT, but not HG, resulted in a significantly stronger N1 in electrodes responsive to speech in the STG. In Figure 3*B*, representative evoked potentials for individual electrodes are shown. The N1 component induced by stimulation of the PT compared with stimulation of the HG was significantly stronger in recorded neural sites in the STG (20.45 ± 0.6 vs 5.7 ± 0.42 μV, *t* test, *p* < 0.001; Fig. 3*C*).

**Figure 4.**
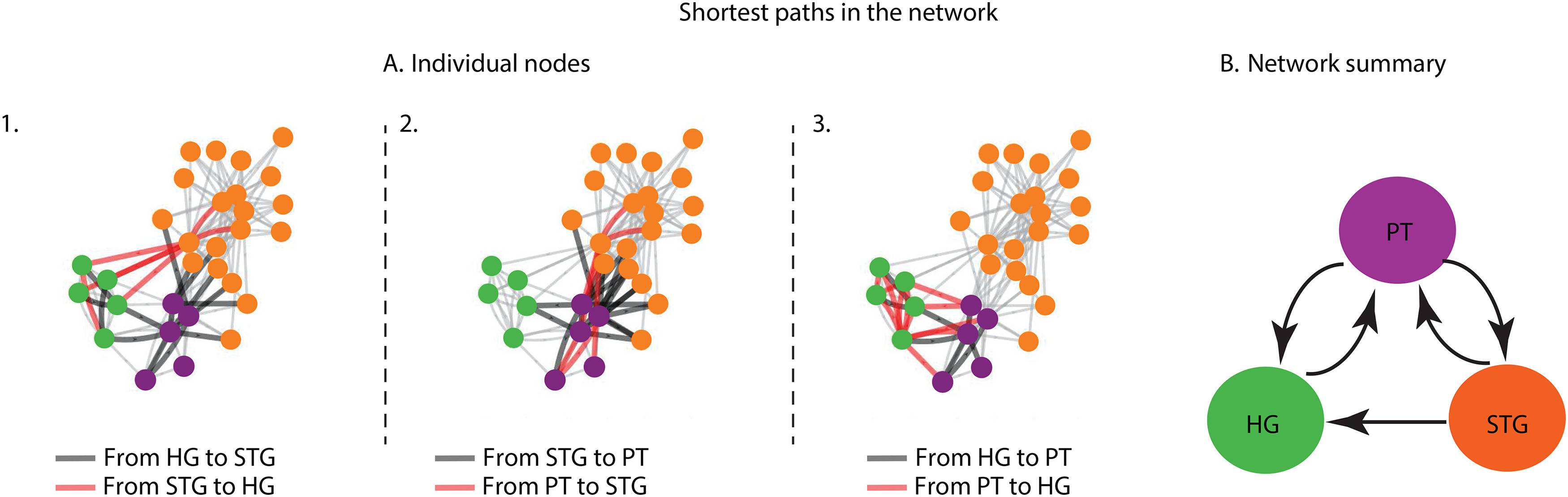
Network connectivity between auditory areas of HG, the PT, and the STG. ***A1***, The shortest path from HG to the STG is shown in black. The shortest path from the STG to HG is shown in red. ***A2***, The shortest path from the PT to the STG is shown in black. The shortest path from the STG to the PT is shown in red. ***A3***, The shortest path from HG to the PT is shown in black. The shortest path from the PT to HG is shown in red. ***B***, Summary of shortest path connectivity.

We generated an adjacency matrix and digraphs to express the connectivity of the neural sites (Biggs et al., 1993). The adjacency matrix of a graph is a square matrix in which the elements indicate the distance between each pair of nodes. From this adjacency matrix, we can generate a digraph (Gross and Yellen, 2005), which is a directed graph of the connectivity between the nodes, with edge arrows showing the direction of the connection and the relative location of nodes showing the strength of connectivity. From the previous analysis, we obtained the CCEPs between two electrodes and normalized their mean and variance based on the neural response before the start of the stimulation. We calculated the absolute value of the N1 component of this normalized CCEP between two electrodes to fill in the elements of the adjacency matrix as a measure of connectivity (rows indicate the stimulated electrode, and columns indicate the target electrode where the CCEP was measured). Thus, each element of this matrix gives a measure of connectivity between the respective electrodes. We further applied a threshold to the adjacency matrix by assigning a value of 1 for significant connections and a value of 0 for nonsignificant connections (see Materials and Methods). Using this adjacency matrix, we generated directional digraph plots with nodes indicating electrodes, edges with arrows indicating the direction of connectivity between the nodes, and the location of nodes based on the method of force-based graph drawing (Fruchterman and Reingold, 1991). In this method, nodes that are not connected are placed further apart in the graph, and those that are highly connected are placed close together in the graph (see Materials and Methods; Fig. 4*A*). This analysis revealed an overall pattern in which the electrodes in HG, the PT, and the STG were placed closer to the electrodes within the same anatomic division, indicating their stronger connectivity (Fig. 4*A*). Moreover, the electrodes in the PT were positioned closer to the electrodes in the STG than to the electrodes in HG, indicating a higher connectivity of the PT sites to the STG than to HG. Furthermore, we measured the shortest path of connectivity between these anatomic groups to estimate the direction of information flow. Since the edges connecting pairs of nodes all have the same weight, the shortest path between two nodes is the path with the smallest number of edges that must be traveled. We found that the direction of connectivity of the HG electrodes to the higher auditory areas of the STG went through the PT. (Fig. 4*A1*). In contrast, the connectivity of PT electrodes to the STG was direct and did not go through HG (Fig. 4*A2*). Finally, the connectivity between the PT and HG was also bidirectional and direct and did not through the STG (Fig. 4*A3*).

A summary of the shortest path analysis is shown in Figure 4*B*. No direct path was found from HG to the STG; instead, the HG sites were connected to the STG sites through the PT sites. Moreover, the electrodes in the PT were placed closer to the electrodes in the STG than to the electrodes in HG. It is important to note that our analysis of the network properties is limited to the electrodes from which we were recording and does not represent all parts of HG or the PT.

**Figure 5.**
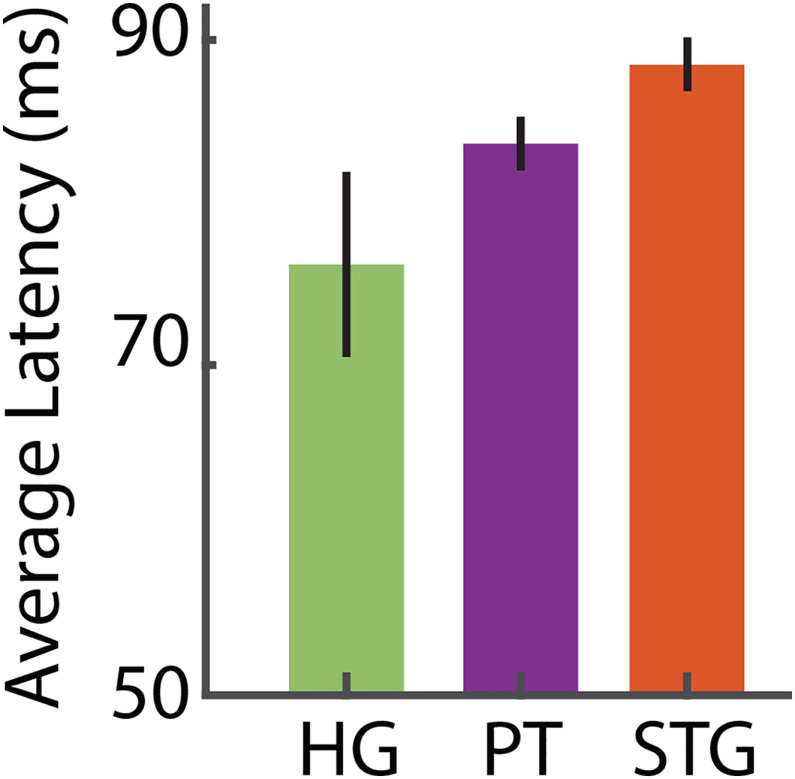
Latency of the neural sites in the HG, the PT, and the STG. The height of bars indicates the mean latency of electrodes in respective anatomic regions. Error bars indicate SE.

The observed intermediate location of the PT is also supported by the results of a response latency analysis (see Materials and Methods). We first calculated the latency of each neural site and averaged the values in each area, as shown in Figure 5. This figure shows that response latency gradually increases from HG to the PT to the STG. The latency of the response along the auditory pathway approximately reflects the number of synapses away from the auditory periphery and hence has been used to speculate the direction of information processing in the auditory cortex (Da Costa et al., 2011; McMurray and Jongman, 2011; Nourski et al., 2014).

### Separation of speech and noise starts in the PT

**Figure 6.**
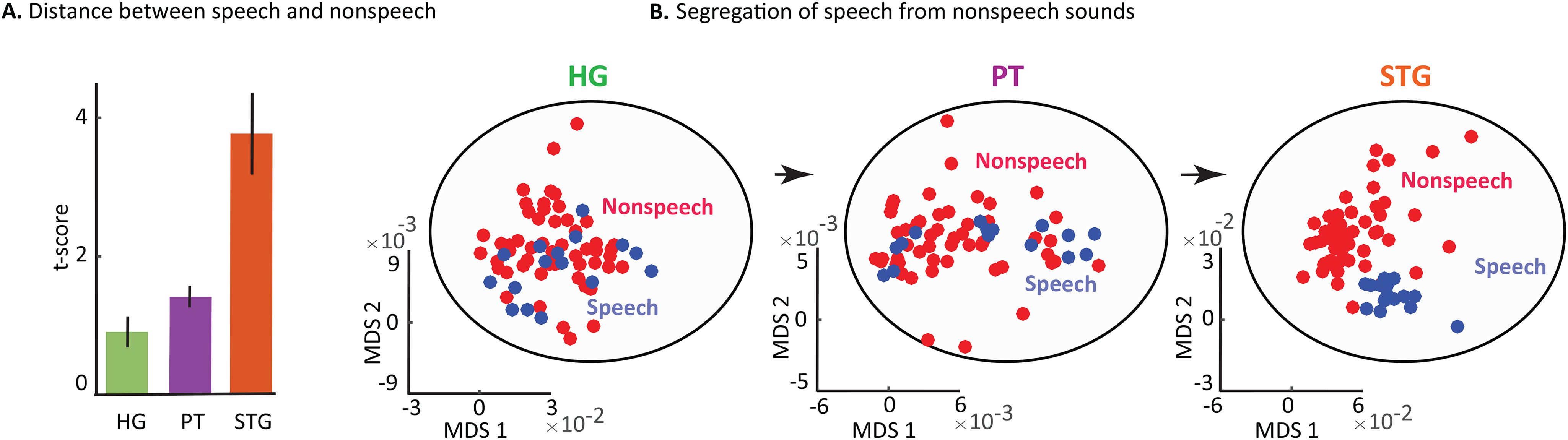
Separation of speech and nonspeech sounds from HG to the PT to the STG. ***A***, The *t* score between speech and nonspeech sounds for each region. Error bars indicate SE. ***B***, Speech versus nonspeech neural responses on an MDS scale.

In addition to latency, we also looked at the separation of speech from nonspeech sounds in each region. Neural responses to specific categories of sounds, such as speech versus nonspeech sounds, have been attributed to a higher level of neural processing in the auditory cortex (Chan et al., 2014). To examine the neural responses to speech versus nonspeech sounds, we designed a task that consisted of 69 samples of sounds: 16 of the sounds were English and foreign news segments, and 53 were diverse sounds, such as coughing, crying, screaming, noise, music, animal vocalization, laughing, singing, shooting, and drumming. We determined the separability of the responses to speech versus nonspeech category of sounds using unpaired *t* test. We observed that the segregation of speech from nonspeech sounds gradually increased from the HG to the PT to the STG (Fig. 6*A*). To better demonstrate how the separation of speech and nonspeech sounds takes place along these regions, we projected the response of sites in each region onto a two-dimensional MDS diagram (see Materials and Methods). Figure 6*B* shows that, while the responses to speech and nonspeech sounds fully overlap in HG, they begin to form separate categories in the PT and become fully separate in the STG.

Our characterization of the electrodes located in HG and the PT and STG can be summarized as follows: (1) CCEP analysis showed that the PT electrodes are situated between the HG and STG electrodes; (2) response latency increases from the HG electrodes to PT electrodes to the STG electrodes; and (3) the responses to speech and nonspeech sounds becomes more distinct from the HG to the PT to the STG electrodes. Collectively, these results suggest that our electrodes in the PT are located between the electrodes in the HG and the STG. This intermediate location might explain the perceptual effect generated by stimulating the electrodes in this region, which is absent during the stimulation of electrodes in HG and the STG.

## Discussion

We report that EBS of electrodes implanted in the PT in one patient significantly and reliably improved both the perceived quality and the intelligibility of speech in noise. The subject reported the suppression of background noise and the selective amplification of speech sounds. We observed significant improvement in both the subjective quality rating and the objective intelligibility score of speech in background noise. The neural sites whose stimulation resulted in enhanced perception of speech had distinct properties compared with the sites in HG; namely, their receptive fields were more tuned to rapid temporal changes in spectrally broad sounds and had a longer response latency. Moreover, network CCEP and speech/nonspeech separability analysis revealed an intermediary role of the neural sites in the PT, suggesting an intermediate functional role relative to the sites in HG and the STG.

Our results causally implicate the examined neural sites in the PT in the suppression of background noise. This triangular region, which occupies the superior temporal plane posterior to HG, is believed to be part of the auditory association cortex (Hickok and Poeppel, 2007). The PT has been suggested to play a role in auditory scene analysis because of its modular structure and multimodal and diverse patterns of neural processing (Griffiths and Warren, 2002). For example, studies have shown that portions of the PT selectively respond to spatial sounds, including moving sounds (Warren et al., 2002; Warren and Griffiths, 2003), with the anterior PT showing sensitivity to pitch-related acoustic changes and the posterior PT showing sensitivity to changes in spatial location (Warren and Griffiths, 2003). Other studies have shown that, in addition to this pure acoustic feature selectivity, the PT is sensitive to spatial sound source locations only when the location serves as a cue for auditory object disambiguation, as opposed to being purely selective of spatial features (Zatorre et al., 2002). Similarly, one study showed that no subregion of the PT was explicitly selective of spatial or object-related information; instead, the PT actively processes spatial cues to aid auditory stream segregation (Smith et al., 2010). Hence, a task/goal-based role of the PT in auditory stream segregation and integration has emerged (Ragert et al., 2014). Consistent with this view, our study is the first to provide experimental evidence in support of an active and causal role of the PT in the crucial task of separating speech from background noise. Moreover, we found that the receptive fields of the PT are distinctly more sensitive to broad spectral temporal changes than the sites in HG. This is in line with anatomic studies showing that the neurite architecture of the PT increases its temporal precision and thus facilitates the processing of auditory speech (Ocklenburg et al., 2018) and with functional studies showing the role of the PT in integrating sequential auditory events (Mustovic et al., 2003). This functional characteristic of the PT may be crucial in auditory scene analysis and noise suppression, as speech and most background noises differ substantially in the spectrotemporal domain (Mesgarani et al., 2006; Mesgarani and Shamma, 2007).

EBS is regarded as complementary to methods that study the neural correlates of stimuli and behavior because EBS directly tests the causal relationship between brain regions and perception. The efficacy of EBS has been demonstrated in vision and memory tasks (Jacobs et al., 2012; Parvizi et al., 2012; Mégevand et al., 2014; Keller et al., 2017; Schalk et al., 2017). Many previous EBS studies relied solely on qualitative descriptions of the subject's experience (Parvizi et al., 2012; Mégevand et al., 2014; Schalk et al., 2017). In contrast, we supplemented the subject's qualitative report with additional quantitative measures to determine the statistical significance of the perceived effects compared with the control (sham) condition. Previous studies of clinical language-mapping protocols used to guide neurosurgical resections have commonly shown the induction of transient language deficits (Borchers et al., 2012). These clinical mapping studies have identified sites that are critical for the perception and discrimination of speech and other complex sounds in the posterior temporal lobe, corresponding to the auditory association cortex (Boatman et al., 1995; Boatman and Miglioretti, 2005), by showing that EBS of the posterior STG causes selective suppression of speech over nonspeech sounds (Sinai et al., 2009). However, to our knowledge, the improvement of speech perception following direct brain stimulation has no precedence in the prior literature. Notably, noninvasive transcranial brain stimulation has also been shown to enhance auditory responses in EEG (Van Doren et al., 2014), improve auditory gap-detection performance (Rufener et al., 2017), bias attention in a spatial multitalker task (Wöstmann et al., 2017), and improve speech perception in cocktail party scenarios (Riecke et al., 2018). Compared with these noninvasive studies, invasive and direct EBS has the power to target a specific brain region, which can result in large and reproducible behavioral effects at the single-trial level, such as those we reported in this study.

Determining how EBS changes neural representation and computation is not a trivial endeavor. It is challenging because of the uncertainty of the spatial extent of the cortex that is responsive to stimulation and the relationship between the circuitry engaged by EBS and the types of neural responses elicited by sensory stimulation. EBS of similar sites in different patients has been shown to yield different results (Borchers et al., 2012; Desmurget et al., 2013). First, small differences in electrode position lead to large differences in targeted responsive neurons (Histed et al., 2009); second, the location of brain functions varies among individual patients (Borchers et al., 2012); and finally, these networks are susceptible to reorganization (Enatsu et al., 2013). Furthermore, EBS can generate complex behavioral effects in regions that are involved in neural processing (Fried et al., 1998; Desmurget et al., 2009; Parvizi et al., 2013). Similar to these studies, we found a specific behavioral effect, although the stimulation signal itself was generic and nonspecific. Additionally, whether EBS of a brain area results in the suppression of or an increase of neural activity is still a matter of scientific debate (Selimbeyoglu and Parvizi, 2010). Extensive preclinical studies have shown that high-frequency (∼100 Hz) EBS increases neuronal excitability (Bliss and Lømo, 1973; Douglas, 1977; Skrede and Malthe-Sørenssen, 1981), whereas low-frequency stimulation (∼1 Hz) decreases neuronal excitability (Mulkey and Malenka, 1992). As a result, several questions warrant further research, including the characterization of local and remote effects of EBS, its inhibitory and excitatory effects (Borchers et al., 2012).

Our study cannot adequately answer how the stimulation of electrodes in the PT results in a perceived reduction in noise. One possible explanation is motivated by our observation of a gradual separation of speech and noise encoding along the auditory pathway (Bar-Yosef and Nelken, 2007; Norman-Haignere et al., 2015). Given these findings, it is possible that the partially separated representations of speech and noise may be differentially targeted by electrical stimulation and hence may be selectively enhanced or suppressed. Consistent with this hypothesis, we found that the representation of speech and noise at PT sites was more separable than the representations at HG sites but less separable than those at STG sites. Additionally, our analysis of latency and network connectivity (CCEP) placed the PT sites between the HG and STG sites. It is plausible that selective suppression of the neural representation of background noise in the PT could reduce its perception. Alternatively, stimulation can activate and excite recurrent connections to upstream and downstream areas that may engage inhibitory connections and hence result in the increased removal of noise from the mixed sound. While we cannot determine the neural mechanism underlying this perceptual effect, future research that allows a better understanding of the role of EBS can result in a more conclusive explanation of the behavioral effect reported in this study and advance our knowledge of the role of the PT in speech-in-noise perception. Finally, speech perception in noise is a very challenging task for individuals with hearing loss. This study opens the possibility of neuroprosthetic solutions that can directly manipulate the cortical representation of speech to enhance the perception of a target sound among competing sources, which can assist individuals who struggle to hear speech in adverse acoustic conditions.

## References

[B1] Assmann P, Summerfield Q (2004) The perception of speech under adverse conditions. In: Speech processing in the auditory system, pp 231–308. Springer: New York, NY.

[B2] Bar-Yosef O, Nelken I (2007) The effects of background noise on the neural responses to natural sounds in cat primary auditory cortex. Front Comput Neurosci 1:3. 10.3389/neuro.10.003.2007 18946525 PMC2525935

[B3] Benjamini Y, Yekutieli D (2001) The control of the false discovery rate in multiple testing under dependency. Annals of statistics 29:1165–1188.

[B4] Biggs N, Biggs NL, Norman B (1993) Algebraic graph theory. Cambridge, MA: Cambridge UP.

[B5] Bliss TV, Lømo T (1973) Long-lasting potentiation of synaptic transmission in the dentate area of the anaesthetized rabbit following stimulation of the perforant path. J Physiol 232:331–356. 10.1113/jphysiol.1973.sp010273 4727084 PMC1350458

[B6] Boatman DF, Miglioretti DL (2005) Cortical sites critical for speech discrimination in normal and impaired listeners. J Neurosci 25:5475–5480. 10.1523/JNEUROSCI.0936-05.2005 15944375 PMC6724973

[B7] Boatman D, Lesser RP, Gordon B (1995) Auditory speech processing in the left temporal lobe: an electrical interference study. Brain Lang 51:269–290. 10.1006/brln.1995.1061 8564472

[B8] Borchers S, Himmelbach M, Logothetis N, Karnath HO (2012) Direct electrical stimulation of human cortex: the gold standard for mapping brain functions? Nat Rev Neurosci 13:63–70. 10.1038/nrn3140 22127300

[B9] Bregman AS (1994) Auditory scene analysis: the perceptual organization of sound. Cambridge, MA: Massachusetts Institute of Technology.

[B10] Brugge JF, Volkov IO, Garell PC, Reale RA, Howard MA III (2003) Functional connections between auditory cortex on Heschl's gyrus and on the lateral superior temporal gyrus in humans. J Neurophysiol 90:3750–3763. 10.1152/jn.00500.2003 12968011

[B11] Buzsáki G, Anastassiou CA, Koch C (2012) The origin of extracellular fields and currents-EEG, ECoG, LFP and spikes. Nat Rev Neurosci 13:407–420. 10.1038/nrn3241 22595786 PMC4907333

[B12] Chan AM, Dykstra AR, Jayaram V, Leonard MK, Travis KE, Gygi B, Baker JM, Eskandar E, Hochberg LR, Halgren E, Cash SS (2014) Speech-specific tuning of neurons in human superior temporal gyrus. Cereb Cortex 24:2679–2693. 10.1093/cercor/bht127 23680841 PMC4162511

[B13] Chi T, Ru P, Shamma SA (2005) Multiresolution spectrotemporal analysis of complex sounds. J Acoust Soc Am 118:887–906. 10.1121/1.1945807 16158645

[B14] Cox MA, Cox TF (2008) Multidimensional scaling. In: Handbook of data visualization, pp 315–347. New York: Springer.

[B15] Da Costa S, van der Zwaag W, Marques JP, Frackowiak RS, Clarke S, Saenz M (2011) Human primary auditory cortex follows the shape of Heschl's gyrus. J Neurosci 31:14067–14075. 10.1523/JNEUROSCI.2000-11.2011 21976491 PMC6623669

[B16] David SV, Mesgarani N, Shamma SA (2007) Estimating sparse spectro-temporal receptive fields with natural stimuli. Network 18:191–212. 10.1080/09548980701609235 17852750

[B17] Desmurget M, Reilly KT, Richard N, Szathmari A, Mottolese C, Sirigu A (2009) Movement intention after parietal cortex stimulation in humans. Science 324:811–813. 10.1126/science.1169896 19423830

[B18] Desmurget M, Song Z, Mottolese C, Sirigu A (2013) Re-establishing the merits of electrical brain stimulation. Trends Cogn Sci 17:442–449. 10.1016/j.tics.2013.07.002 23932195

[B19] Donovan C, Sweet J, Eccher M, Megerian C, Semaan M, Murray G, Miller J (2015) Deep brain stimulation of Heschl gyrus: implantation technique, intraoperative localization, and effects of stimulation. Neurosurgery 77:940–947. 10.1227/NEU.0000000000000969 26280116

[B20] Douglas RM (1977) Long lasting synaptic potentiation in the rat dentate gyrus following brief high frequency stimulation. Brain Res 126:361–365. 193611 10.1016/0006-8993(77)90733-8

[B21] Edwards E, Soltani M, Kim W, Dalal SS, Nagarajan SS, Berger MS, Knight RT (2009) Comparison of time–frequency responses and the event-related potential to auditory speech stimuli in human cortex. J Neurophysiol 102:377–386. 10.1152/jn.90954.2008 19439673 PMC2712274

[B22] Elmer S, Hänggi J, Meyer M, Jäncke L (2013) Increased cortical surface area of the left planum temporale in musicians facilitates the categorization of phonetic and temporal speech sounds. Cortex 49:2812–2821. 10.1016/j.cortex.2013.03.007 23628644

[B23] Enatsu R, Kubota Y, Kakisaka Y, Bulacio J, Piao Z, O'Connor T, Horning K, Mosher J, Burgess RC, Bingaman W, Nair DR (2013) Reorganization of posterior language area in temporal lobe epilepsy: a cortico-cortical evoked potential study. Epilepsy Res 103:73–82. 10.1016/j.eplepsyres.2012.07.008 22819071

[B24] Fenoy AJ, Severson MA, Volkov IO, Brugge JF, Howard MA III (2006) Hearing suppression induced by electrical stimulation of human auditory cortex. Brain Res 1118:75–83. 10.1016/j.brainres.2006.08.013 16979144 PMC3816378

[B25] Fried I, Wilson CL, MacDonald KA, Behnke EJ (1998) Electric current stimulates laughter. Nature 391:650. 10.1038/35536 9490408

[B26] Fruchterman TM, Reingold EM (1991) Graph drawing by force-directed placement. Softw: Pract Exp 21:1129–1164. 10.1002/spe.4380211102

[B27] Griffiths TD, Warren JD (2002) The planum temporale as a computational hub. Trends Neurosci 25:348–353. 10.1016/S0166-2236(02)02191-4 12079762

[B28] Griffiths TD, Warren JD (2004) What is an auditory object? Nat Rev Neurosci 5:887–892. 10.1038/nrn1538 15496866

[B29] Gross JL, Yellen J (2005) Graph theory and its applications. Boca Raton, FL: CRC.

[B30] Hickok G, Poeppel D (2007) The cortical organization of speech processing. Nat Rev Neurosci 8:393–402. 10.1038/nrn2113 17431404

[B31] Hickok G, Saberi K (2012) Redefining the functional organization of the planum temporale region: space, objects, and sensory–motor integration. In: The human auditory cortex, pp 333–350. New York: Springer.

[B32] Histed MH, Bonin V, Reid RC (2009) Direct activation of sparse, distributed populations of cortical neurons by electrical microstimulation. Neuron 63:508–522. 10.1016/j.neuron.2009.07.016 19709632 PMC2874753

[B33] Hong S, Lundstrom BN, Fairhall AL (2008) Intrinsic gain modulation and adaptive neural coding. PLoS Comput Biol 4:e1000119. 10.1371/journal.pcbi.1000119 18636100 PMC2440820

[B34] Humphries C, Sabri M, Lewis K, Liebenthal E (2014) Hierarchical organization of speech perception in human auditory cortex. Front Neurosci 8:406. 10.3389/fnins.2014.00406 25565939 PMC4263085

[B35] Isenberg AL, Vaden KI Jr, Saberi K, Muftuler LT, Hickok G (2012) Functionally distinct regions for spatial processing and sensory motor integration in the planum temporale. Hum Brain Mapp 33:2453–2463. 10.1002/hbm.21373 21932266 PMC5242090

[B36] Jacobs J, Lega B, Anderson C (2012) Explaining how brain stimulation can evoke memories. J Cogn Neurosci 24:553–563. 10.1162/jocn_a_00170 22098266

[B37] Kell AJ, McDermott JH (2019) Invariance to background noise as a signature of non-primary auditory cortex. Nat Commun 10:3958. 31477711 10.1038/s41467-019-11710-y PMC6718388

[B38] Keller CJ, Bickel S, Entz L, Ulbert I, Milham MP, Kelly C, Mehta AD (2011) Intrinsic functional architecture predicts electrically evoked responses in the human brain. Proceedings of the National Academy of Sciences 108:10308–10313. 10.1073/pnas.1019750108 PMC3121855 21636787

[B39] Keller CJ, Honey CJ, Mégevand P, Entz L, Ulbert I, Mehta AD (2014a) Mapping human brain networks with cortico-cortical evoked potentials. Philos Trans R Soc Lond B Biol Sci 369:20130528. 10.1098/rstb.2013.0528 25180306 PMC4150303

[B40] Keller CJ, Honey CJ, Entz L, Bickel S, Groppe DM, Toth E, Ulbert I, Lado FA, Mehta AD (2014b) Corticocortical evoked potentials reveal projectors and integrators in human brain networks. J Neurosci 34:9152–9163. 10.1523/JNEUROSCI.4289-13.2014 24990935 PMC4078089

[B41] Keller CJ, Davidesco I, Megevand P, Lado FA, Malach R, Mehta AD (2017) Tuning face perception with electrical stimulation of the fusiform gyrus. Hum Brain Mapp 38:2830–2842. 10.1002/hbm.23543 28345189 PMC5426961

[B42] Keller CJ, Huang Y, Herrero JL, Fini ME, Du V, Lado FA, Honey CJ, Mehta AD (2018) Induction and quantification of excitability changes in human cortical networks. J Neurosci 38:5384–5398. 10.1523/JNEUROSCI.1088-17.2018 29875229 PMC5990984

[B43] Khalighinejad B, Nagamine T, Mehta A, Mesgarani N (2017) NAPLib: an open source toolbox for real-time and offline Neural Acoustic Processing. Acoustics, Speech and Signal Processing, 2017 IEEE International Conference, pp 846–850. 10.1109/ICASSP.2017.7952275 PMC5805377 29430213

[B44] Khalighinejad B, Herrero JL, Mehta AD, Mesgarani N (2019) Adaptation of the human auditory cortex to changing background noise. Nat Commun 10:2509. 31175304 10.1038/s41467-019-10611-4 PMC6555798

[B45] Khalighinejad B, Patel P, Herrero JL, Bickel S, Mehta AD, Mesgarani N (2021) Functional characterization of human Heschl's gyrus in response to natural speech. Neuroimage 235:118003. 10.1016/j.neuroimage.2021.118003 33789135 PMC8608271

[B46] Kumar S, Stephan KE, Warren JD, Friston KJ, Griffiths TD (2007) Hierarchical processing of auditory objects in humans. PLoS Comput Biol 3:e100. 10.1371/journal.pcbi.0030100 17542641 PMC1885275

[B47] Leonard MK, Cai R, Babiak MC, Ren A, Chang EF (2019) The peri-Sylvian cortical network underlying single word repetition revealed by electrocortical stimulation and direct neural recordings. Brain Lang 193:58–72. 10.1016/j.bandl.2016.06.001 27450996 PMC5790638

[B48] McMurray B, Jongman A (2011) What information is necessary for speech categorization? Harnessing variability in the speech signal by integrating cues computed relative to expectations. Psychol Rev 118:219–246. 10.1037/a0022325 21417542 PMC3523696

[B49] Mégevand P, Groppe DM, Goldfinger MS, Hwang ST, Kingsley PB, Davidesco I, Mehta AD (2014) Seeing scenes: topographic visual hallucinations evoked by direct electrical stimulation of the parahippocampal place area. J Neurosci 34:5399–5405. 10.1523/JNEUROSCI.5202-13.2014 24741031 PMC6608225

[B50] Mesgarani N, Shamma S (2007) Denoising in the domain of spectrotemporal modulations. EURASIP J 3:1–8.

[B51] Mesgarani N, Slaney M, Shamma SA (2006) Discrimination of speech from nonspeech based on multiscale spectro-temporal modulations. IEEE Trans Audio Speech Lang Process 14:920–930. 10.1109/TSA.2005.858055

[B52] Mesgarani N, David SV, Fritz JB, Shamma SA (2014) Mechanisms of noise robust representation of speech in primary auditory cortex. Proc Natl Acad Sci USA 111:6792–6797. 10.1073/pnas.1318017111 24753585 PMC4020083

[B53] Meyer M, Elmer S, Jäncke L (2012) Musical expertise induces neuroplasticity of the planum temporale. Ann NY Acad Sci 1252:116–123. 10.1111/j.1749-6632.2012.06450.x 22524348

[B54] Miglioretti DL, Boatman D (2003) Modeling variability in cortical representations of human complex sound perception. Exp Brain Res 153:382–387. 10.1007/s00221-003-1703-2 14534769

[B55] Miller LM, Escabı MA, Read HL, Schreiner CE (2001) Functional convergence of response properties in the auditory thalamocortical system. Neuron 32:151–160. 10.1016/S0896-6273(01)00445-7 11604146

[B56] Morosan P, Rademacher J, Palomero-Gallagher N, Zilles K (2005) Anatomical organization of the human auditory cortex: cytoarchitecture and transmitter receptors. In: The auditory cortex, pp 45–68. Hove, East Sussex, United Kingdom: Psychology Press.

[B57] Mulkey RM, Malenka RC (1992) Mechanisms underlying induction of homosynaptic long-term depression in area CA1 of the hippocampus. Neuron 9:967–975. 10.1016/0896-6273(92)90248-C 1419003

[B58] Mustovic H, Scheffler K, Di Salle F, Esposito F, Neuhoff JG, Hennig J, Seifritz E (2003) Temporal integration of sequential auditory events: silent period in sound pattern activates human planum temporale. Neuroimage 20:429–434. 10.1016/S1053-8119(03)00293-3 14527603

[B59] Norman-Haignere S, Kanwisher NG, McDermott JH (2015) Distinct cortical pathways for music and speech revealed by hypothesis-free voxel decomposition. Neuron 88:1281–1296. 10.1016/j.neuron.2015.11.035 26687225 PMC4740977

[B60] Nourski KV, Steinschneider M, McMurray B, Kovach CK, Oya H, Kawasaki H, Howard MA (2014) Functional organization of human auditory cortex: investigation of response latencies through direct recordings. Neuroimage 101:598–609. 10.1016/j.neuroimage.2014.07.004 25019680 PMC4430832

[B61] Ocklenburg S, Friedrich P, Fraenz C, Schlüter C, Beste C, Güntürkün O, Genç E (2018) Neurite architecture of the planum temporale predicts neurophysiological processing of auditory speech. Sci Adv 4:eaar6830. 10.1126/sciadv.aar6830 30009258 PMC6040861

[B62] Parvizi J, Kastner S (2018) Promises and limitations of human intracranial electroencephalography. Nat Neurosci 21:474–483. 10.1038/s41593-018-0108-2 29507407 PMC6476542

[B63] Parvizi J, Jacques C, Foster BL, Witthoft N, Rangarajan V, Weiner KS, Grill-Spector K (2012) Electrical stimulation of human fusiform face-selective regions distorts face perception. J Neurosci 32:14915–14920. 10.1523/JNEUROSCI.2609-12.2012 23100414 PMC3517886

[B64] Parvizi J, Rangarajan V, Shirer WR, Desai N, Greicius MD (2013) The will to persevere induced by electrical stimulation of the human cingulate gyrus. Neuron 80:1359–1367. 10.1016/j.neuron.2013.10.057 24316296 PMC3877748

[B65] Rabinowitz NC, Willmore BD, King AJ, Schnupp JW (2013) Constructing noise-invariant representations of sound in the auditory pathway. PLoS Biol 11:e1001710. 10.1371/journal.pbio.1001710 24265596 PMC3825667

[B66] Ragert M, Fairhurst MT, Keller PE (2014) Segregation and integration of auditory streams when listening to multi-part music. PLoS One 9:e84085. 10.1371/journal.pone.0084085 24475030 PMC3901649

[B67] Ray S, Maunsell JH (2011) Different origins of gamma rhythm and high-gamma activity in macaque visual cortex. PLoS Biol 9:e1000610. 10.1371/journal.pbio.1000610 21532743 PMC3075230

[B68] Riecke L, Formisano E, Sorger B, Başkent D, Gaudrain E (2018) Neural entrainment to speech modulates speech intelligibility. Curr Biol 28:161–169. 10.1016/j.cub.2017.11.033 29290557

[B69] Rufener KS, Ruhnau P, Heinze HJ, Zaehle T (2017) Transcranial random noise stimulation (tRNS) shapes the processing of rapidly changing auditory information. Front Cell Neurosci 11:162. 10.3389/fncel.2017.00162 28642686 PMC5463504

[B70] Salza PL, Foti E, Nebbia L, Oreglia M (1996) MOS and pair comparison combined methods for quality evaluation of text-to-speech systems. Acta Acust. United with Acust 82:650–656.

[B71] Schalk G, Kapeller C, Guger C, Ogawa H, Hiroshima S, Lafer-Sousa R, Saygin ZM, Kamada K, Kanwisher N (2017) Facephenes and rainbows: causal evidence for functional and anatomical specificity of face and color processing in the human brain. Proc Natl Acad Sci USA 114:12285–12290. 29087337 10.1073/pnas.1713447114 PMC5699078

[B72] Selimbeyoglu A, Parvizi J (2010) Electrical stimulation of the human brain: perceptual and behavioral phenomena reported in the old and new literature. Front Hum Neurosci 4:46. 10.3389/fnhum.2010.00046 20577584 PMC2889679

[B73] Sinai A, Crone NE, Wied HM, Franaszczuk PJ, Miglioretti D, Boatman-Reich D (2009) Intracranial mapping of auditory perception: event-related responses and electrocortical stimulation. Clin Neurophysiol 120:140–149. 10.1016/j.clinph.2008.10.152 19070540 PMC2819074

[B74] Sinha SR, Crone NE, Fotta R, Lenz F, Boatman DF (2005) Transient unilateral hearing loss induced by electrocortical stimulation. Neurology 64:383–385. 10.1212/01.WNL.0000149524.11371.B1 15668450

[B75] Skrede KK, Malthe-Sørenssen D (1981) Increased resting and evoked release of transmitter following repetitive electrical tetanization in hippocampus: a biochemical correlate to long-lasting synaptic potentiation. Brain Res 208:436–441. 10.1016/0006-8993(81)90573-4 6260292

[B76] Smith KR, Hsieh IH, Saberi K, Hickok G (2010) Auditory spatial and object processing in the human planum temporale: no evidence for selectivity. J Cogn Neurosci 22:632–639. 10.1162/jocn.2009.21196 19301992

[B77] Theunissen FE, David SV, Singh NC, Hsu A, Vinje WE, Gallant JL (2001) Estimating spatio-temporal receptive fields of auditory and visual neurons from their responses to natural stimuli. Netw Comput Neural Syst 12:289–316. 10.1080/net.12.3.289.316 11563531

[B78] Van Doren J, Langguth B, Schecklmann M (2014) Electroencephalographic effects of transcranial random noise stimulation in the auditory cortex. Brain Stimul 7:807–812. 25245591 10.1016/j.brs.2014.08.007

[B79] Wang K, Shamma S (1994) Self-normalization and noise-robustness in early auditory representations. Speech Audio Process IEEE Trans 2:421–435. 10.1109/89.294356

[B80] Warren JD, Griffiths TD (2003) Distinct mechanisms for processing spatial sequences and pitch sequences in the human auditory brain. J Neurosci 23:5799–5804. 10.1523/JNEUROSCI.23-13-05799.2003 12843284 PMC6741275

[B81] Warren JD, Zielinski BA, Green GG, Rauschecker JP, Griffiths TD (2002) Perception of sound-source motion by the human brain. Neuron 34:139–148. 10.1016/S0896-6273(02)00637-2 11931748

[B82] Wilson RH, McArdle RA, Smith SL (2007) An evaluation of the BKB-SIN, HINT, QuickSIN, and WIN materials on listeners with normal hearing and listeners with hearing loss. J Speech Lang Hear Res 50:844–856. 10.1044/1092-4388(2007/059) 17675590

[B83] Wöstmann M, Vosskuhl J, Obleser J, Herrmann C (2017) Opposite effects of lateralised transcranial alpha versus gamma stimulation on spatial attention. bioRxiv 180836. 10.1016/j.brs.2018.04.006 29656907

[B84] Zatorre RJ, Bouffard M, Ahad P, Belin P (2002) Where is 'where' in the human auditory cortex? Nat Neurosci 5:905–909. 10.1038/nn904 12195426

